# *Health Inequality Monitoring* channel on OpenWHO: capacity strengthening through eLearning

**DOI:** 10.1186/s12939-022-01739-9

**Published:** 2022-09-13

**Authors:** Nicole Bergen, Katherine Kirkby, Andreia Baptista, Devaki Nambiar, Anne Schlotheuber, Cecilia Vidal Fuertes, Ahmad Reza Hosseinpoor

**Affiliations:** Department of Data and Analytics, Division of Data, Analytics and Delivery for Impact, World Health Organization, 20, Avenue Appia, CH-1211 Geneva 27, Switzerland

## Abstract

**Background:**

Health inequality monitoring can generate important evidence to inform and motivate changes to policy, programmes and practices. However, the potential of health inequality monitoring practices to quantify inequalities between population subgroups and track progress on the advancement of health equity is under-realized. Capacity strengthening on health inequality monitoring can play an important role in enhancing political will for the generation and use of disaggregated data and for wider adoption of this practice to inform health decision-making. There is a lack of widely available and accessible training materials related to health inequality monitoring that may be used by a range of stakeholders.

**Objective:**

In this paper, we describe the design, development and implementation of the *Health Inequality Monitoring* channel on the OpenWHO eLearning platform. We discuss the anticipated impact and potential opportunities for these eLearning courses to contribute to strengthened health inequality monitoring practices.

**Results:**

The *Health Inequality Monitoring* channel on the OpenWHO platform is a self-directed learning environment, designed to meet the immediate learning needs of users. The channel contains three series of courses: health inequality monitoring foundations courses; topic-specific health inequality monitoring courses; and health inequality monitoring skill building courses. Courses are primarily targeted to monitoring and evaluation officers, data analysts, academics and researchers, public health professionals, medical and public health students, and others with a general interest in health data and inequality monitoring.

**Conclusions:**

WHO eLearning courses on health inequality monitoring are addressing the need for capacity strengthening in the collection, analysis and reporting of inequality data. They introduce learners to the foundational concepts, best practices, tools and skills required to conduct health inequality monitoring. The courses on the *Health Inequality Monitoring* channel demonstrate how technical information can be simplified and presented to broad audiences in a manner that is highly accessible to learners. The *Health Inequality Monitoring* channel on OpenWHO is an innovative and necessary addition to existing tools and resources to support the advancement of health equity.

## Background

The advancement of health equity is a stated priority across diverse health programs and development initiatives. Notably, equity is embedded in the 17 global goals laid out in the United Nations 2030 Agenda for Sustainable Development, which pledges to leave no one behind. Goal 3, to ensure healthy lives and promote well-being for all at all ages, includes a commitment to advancing access to quality essential health care services, medicines and vaccines, with financial risk protection, through universal health coverage [1].

The World Health Organization (WHO) describes health equity as the absence of unfair, avoidable and remediable differences in health status among groups of people [2, 3]. Health inequalities are measurable differences in health across population subgroups. Health inequality monitoring, the process of quantifying and assessing health inequalities in a defined population, is critical to determine where changes to policies, programmes and practices are needed to advance health equity [4].

As part of its work to promote health equity, WHO recognizes the need for strengthening country capacity for monitoring and analysis using disaggregated data [5, 6]. Disaggregated health data are vital to identify where and why health inequalities exist, as they enable comparisons of how health experiences vary across population groups defined by sex, age, economic status, education level, place of residence, and other factors. Yet, only half of countries include disaggregation in national health statistics reports, according to the 2020 WHO SCORE Global Report, which assessed the status and capacity of health information systems across 133 countries [7]. Therefore, the potential of health inequality monitoring is under-realized.

The WHO Health Inequality Monitoring team aims to strengthen capacity for health inequality monitoring among Member States. The team generates and disseminates the latest evidence on health inequalities, and advances approaches for inequality monitoring, including developing and refining tools and resources. To this end, the team has developed numerous resources with complementary applications, all contributing to an overarching vision of advancing equity through the improved use of data by monitoring health inequality (Table 1).

**Table 1:** Selected WHO tools and resources for health inequality monitoring

Resource	Description	Purpose
Health Inequality Data Repository [8]	This large repository of disaggregated data represents a variety of health topics, themes and settings. It includes datasets of country indicators, global datasets about select health topics and global datasets about select determinants of health	The data repository facilitates access to a large bank of disaggregated data for health inequality analyses. The data can be explored interactively online and are available for download
Health Equity Assessment Toolkit (HEAT and HEAT Plus) [9]	This interactive application allows users to explore patterns of inequality, calculate summary measures of inequality and create and export customized graphs, maps and tables. HEAT has a built-in database, while HEAT Plus allows users to upload and work with their own data	HEAT and HEAT Plus is a user-friendly tool designed to facilitate the analysis, interpretation and reporting of disaggregated data and summary measures of health inequality. It allows users to synthesize and visualize data on the status of health inequality in a setting of interest and compare across similar settings
Health inequality monitoring capacity building workshops	In-person and online workshops, conducted with participants from WHO Member States and Regions, explore the theoretical and practical aspects of health inequality monitoring. The workshop materials and datasets are tailored to the needs and priorities of participating countries Complementary Training-of-Trainers (TOT) sessions encourage further knowledge dissemination through subsequent workshops	Capacity building workshops promote national health inequality monitoring practices and provide an environment for networking among participants. These workshops provide participants with the skills to implement or strengthen health inequality monitoring systems, including interpreting disaggregated data and basic summary measures. Participating countries are encouraged to prepare post-workshop ‘state of inequality’ reports and promote health inequality monitoring within their countries and regions
Handbook on health inequality monitoring: with a special focus on low- and middle-income countries [4]	The Handbook outlines theoretical concepts related to health inequality monitoring, and elaborates on a stepwise monitoring process, drawing examples from low- and middle-income countries	The Handbook is primarily designed for technical staff of ministries of health as a reference for establishing and strengthening national inequality monitoring systems
Step-by-step manuals [10–12]	A series of step-by-step manuals for health inequality monitoring provides practical guidance on how to carry out the five-step cycle of health inequality monitoring in the context of national monitoring, as well as specific topics. The five-step cycle covers: (a) determining the scope of monitoring; (b) obtaining data; (c) analysing data; (d) reporting results; and (e) translating knowledge into action	The manuals are designed as highly accessible, practical references for health inequality monitoring. They are each organized according to the five steps, with key questions and checklists for each substep, and examples, references, best practices, and glossaries
Workbooks [13, 14]	Workbooks contain exercises that facilitate the application of the general concepts of health inequality monitoring, including questions and prompts to make decisions at each step of monitoring, as well as table templates	The workbooks can be used as detailed internal records of the monitoring process and the rationale for how it was undertaken
‘State of inequality’ and ‘Explorations of inequality’ reports [15–19]	This series comprises detailed reports of health inequalities, using disaggregated health data to analyze the latest situation of inequality and changes in inequality over time, contextualized within the current state of knowledge from the broader literature. They discuss the implications of findings, provide examples of approaches to address unfair and remediable inequalities, and present opportunities for strengthening inequality monitoring. The reports integrate digital data visualization technology to present data interactively	These are examples of high-quality health inequality reporting, targeted to audiences with expertise or interest in the featured topic area or setting. In several cases, the reports represent the first systematic global assessment of inequalities in the topic area or setting

To complement and enhance the impact of existing tools and resources, the Health Inequality Monitoring team has developed an eLearning channel dedicated to advancing knowledge and skills pertaining to health inequality monitoring. The courses are complementary to existing tools and resources in terms of their content, for example, elaborating upon components of the five-step cycle of monitoring, highlighting examples from inequality reports and the Health Inequality Data Repository, and referring learners to Health Equity Assessment Toolkit (HEAT). The online courses are learner oriented, providing accessibility to global audiences and allowing for flexibility through self-directed learning.

The Health Inequality Monitoring team launched the *Health Inequality Monitoring* channel as part of the worldwide social learning network, OpenWHO [20]. OpenWHO offers free, interactive, online courses to global audiences, featuring multiple health topics and delivering courses in numerous languages. The platform has reported course completion rates of over 45%, with nearly half of learners having enrolled in at least two courses and over 70,000 learners having completed ten or more courses [21]. Between 2019 and 2021 (during the COVID-19 pandemic), the use of the platform increased by previously underrepresented groups including women and younger and older learners [22]. The *Health Inequality Monitoring* channel will contain a collection of eLearning courses about the foundations of health inequality monitoring, its application to specific topics and skill building. These courses are initially available in English with written transcripts of audio and audio-visual components; language translations and subtitles may be integrated into course updates.

In this article, we describe the development of the *Health Inequality Monitoring* channel as well as its features and contents. We will examine how these health inequality monitoring eLearning courses serve as an innovative and necessary addition to support the advancement of health equity.

## Course design, development and implementation

The Health Inequality Monitoring team followed a process outlined in the Analysis-Design-Development-Implement-Evaluate (ADDIE) model for developing instructional courses and training programmes [23]. This model, which is highly adaptable across settings and applications, emphasizes a learner-based approach that relies on the continuous evolution of courses through feedback from diverse stakeholders. It has been previously applied in the development of eLearning courses for the public health and health care workforce, generating a series of best practices [24] that benefited the development of the health inequality monitoring courses.

The analysis phase included the initial conceptualization of the health inequality monitoring eLearning courses and the assessment of learner characteristics and knowledge gaps. Course contents were partly derived from the team’s reports, tools and resources, which included inputs from subject-matter experts. This phase was also informed by the experiences of the Health Inequality Monitoring team in conducting health inequality monitoring capacity building workshops over the past 15 years (see Table 1).^1^ Each workshop gathered feedback from workshop participants through a short post-workshop survey. Interactions with workshop participants across different settings provided rich insights into their knowledge, skills, experiences, and technical abilities, as well as their interests and needs with regards to health inequality monitoring. A particular need emerged for a learning option that was highly accessible, flexible and complementary with existing resources. The team reviewed various eLearning platforms as options to host the courses and compiled a list of their respective functionalities. The team consulted scientific and grey literature about eLearning best practices across different contexts to determine the general features that would enhance the impact of the courses.

As part of the design phase, the Health Inequality Monitoring team brainstormed an initial list of topics to address expressed learner interests and capacity-strengthening opportunities. These topics were grouped by theme (constituting individual courses comprised of modules) and further organized according to their intended purpose (constituting a series). In preparing course applications for the eLearning platform, the team identified preliminary learning objectives for the courses and constituent modules. This process entailed mapping several courses simultaneously and reviewing the scope and content of each to ensure compatibility and complementarity. The team also identified the course presentation style; formative and summative assessment approaches; and learner certificate requirements.

The development phase involved the preparation of course transcripts, visual and audio components, quiz questions, additional reading lists, examples, exercises and other accompanying materials. These materials underwent reviews by the Health Inequality Monitoring team as well as, where appropriate, reviews by WHO staff members and consultants, subject matter experts, and learners. The feedback solicited from the reviewers helped to enhance and refine diverse aspects of the courses. WHO staff members and consultants provided technical reviews and ensured consistency and linkages with existing and planned WHO tools and resources, including those for health inequality monitoring. Senior-level WHO colleagues reviewed select course materials, as warranted, to advise on potential sensitivities. Subject matter experts provided technical reviews, ensuring that the learning material reflects the current state of knowledge in the field. Learner perspectives and feedback enhanced the usability and clarity of the course materials.

During the implement phase, the OpenWHO team uploaded the course materials to the OpenWHO eLearning platform. Testing was carried out by members of the Health Inequality Monitoring team before publication. Concurrent with the publication of the eLearning courses, the Health Inequality Monitoring team held virtual launch events to promote their wide dissemination. Although the evaluate phase was undertaken, in part, within the course development, a process was integrated for obtaining continuous feedback about the eLearning courses directly from learners using linear scale and free text questions. Learner feedback will assist in the ongoing improvement and planned periodic updates of the courses.

### *Health inequality monitoring* channel

Health inequality monitoring eLearning courses address an unmet need for a self-directed learning environment where learners can acquire a conceptual understanding of the monitoring process, navigate its application to different health topics, and learn/refine practical skills. The *Health Inequality Monitoring* channel therefore contains three distinct series of courses. Health inequality monitoring foundations courses introduce the components of health inequality monitoring, emphasizing key concepts and best practices. Topic-specific health inequality monitoring courses showcase the application of health inequality monitoring within specific health topics. Health inequality monitoring skill building courses provide practical guidance on health inequality analysis methods, including the use of selected software programmes. The target audiences for the courses on the *Health Inequality Monitoring *channel include monitoring and evaluation officers, data analysts, academics and researchers, public health professionals, medical and public health students, and others with a general interest in health data, inequality monitoring and data analysis. Learners can be from individual countries (including in ministries of health and other health institutes, statistical offices, universities and non-governmental organisations) or working at thein multilateral or inter-governmental organizations (such as agencies of the United Nations, including WHO).

The OpenWHO eLearning platform was selected to host these courses. OpenWHO contains many courses related to a variety of public health topics, therefore accommodating a large and varied audience, including the target audience for health inequality monitoring courses. The functionalities of the platform met the requirements of the team, including: free access to online courses; straightforward user interface; multiple evaluation (quiz question) formats and assessment options; discussion forums and collab spaces; and certificates of achievement. In addition, the platform allowed for: upload of accompanying learning materials; creation of a dedicated thematic channel; tracking of course enrollment, learner engagement and feedback; regular course updates; addition of new courses to the channel; and translation of courses into multiple languages.

An initial set of 12 courses was planned for the *Health Inequality Monitoring* channel. This included five health inequality monitoring foundations courses, launched in September 2022. Each course is approximately one and a half hours in duration, with the content delivered through 4–6 modules (Appendix 1). The *Overview* course gives a general introduction to the monitoring process and key terminology and concepts, highlighting how it can be adapted to different contexts. It serves as an entry point for the other courses in this series. The *Data sources* course examines the strengths, limitations and opportunities to improve common data sources for health inequality monitoring. It covers household surveys, administrative data sources, civil registration and vital statistics systems and censuses, as well as the processes of data source mapping and data linking. The *Health data disaggregation* course explores how disaggregated health data are integral across the steps of monitoring, helping learners gain skills in assessing and reporting disaggregated data. The *Summary measures of health inequality* course breaks down the general characteristics of simple and complex summary measures, and guides learners through the selection, calculation, interpretation and reporting of a range of measures. The *Reporting* course addresses the components of high-quality health inequality reporting, emphasizing purpose-driven, audience-centred, and technically rigorous approaches. It includes a set of best practices for reporting health inequalities.

Two topic-specific health inequality monitoring courses have been launched, each two hours in duration (Appendix 2). The *Inequality monitoring in immunization* course (launched in December 2021) and *Inequality monitoring in HIV, tuberculosis and malaria* course (launched in May 2022) examine the five general steps of inequality monitoring in the context of immunization programmes as well as HIV, tuberculosis and malaria programmes, respectively. A third course in this series, *Inequality monitoring in sexual, reproductive, maternal, newborn, child and adolescent health*, is under development.

The skill building course series consists of four planned courses, addressing the use of HEAT and HEAT Plus, and the use of software programmes for health inequality analyses (featuring Excel, R and Stata). These courses, anticipated for launch in December 2022, are each two hours in duration.

## Anticipated impact and opportunities

The *Health Inequality Monitoring* channel on OpenWHO is an innovative and necessary addition to existing tools and resources to support the advancement of health equity. To our knowledge, these courses constitute the most comprehensive online collection of eLearning courses to build capacity in health inequality monitoring. Given that the reduction of inequities is a common aim across prominent global health and development strategies, the need for capacity building for health inequality monitoring is widespread and dispersed.

The *Health Inequality Monitoring* channel is an opportunity to reach global audiences who have a range of learning interests and needs. The concepts and skills covered in the courses can be applied to diverse health topics, across different types of settings (including centralized and decentralized levels of the health sector), for multiple purposes, and by diverse stakeholders.

Certain features of the *Health Inequality Monitoring* channel serve to attract learners and enhance their retention, engagement, motivation and outcomes. Courses are offered for free to promote participation. Course materials are available in multiple formats and can be downloaded and used offline, thereby increasing accessibility to the courses in contexts with low bandwidth. Learners have the option to selectively access course modules of interest without navigating through the entire course or course series. This helps to ensure that the courses meet their immediate learning needs. Cross-referencing between courses provides further direction for learners to access relevant material and helps learners understand how the content of different courses is linked. For certain courses, exercises and worksheets are provided to situate learning and help learners develop and practice key competencies (for example, skill building courses on the use of statistical programmes provide sample datasets and codes). Discussion forums and collaboration spaces are available for learners to pose questions and interact with other learners, providing an opportunity for joint learning and networking. Learners can earn a certificate of achievement by scoring at least 80% on the graded final assessment of the course (non-graded assessments are also offered for each module so that learners can gage their level of understanding throughout the course). Certificates can be shared as a verified badge through social media or email.

The courses on the *Health Inequality Monitoring* channel are intended for use in concert with other health inequality monitoring tools and resources. The eLearning courses were designed around the same cycle of monitoring and principles as other resources by the Health Inequality Monitoring team. For instance, the courses draw examples of best practices from ‘State of inequality’ reports and showcase the use of tools such as HEAT. The topic-specific applications courses complement published thematic reports or step-by-step manuals. Forthcoming health inequality monitoring capacity building workshops by the Health Inequality Monitoring team will require completion of some or all of the health inequality monitoring foundations courses as a prerequisite to participation in the workshop. This is anticipated to support learner retention of knowledge and allow for deeper engagement surrounding the application of concepts during the workshop.

Launch events to mark the release of courses on the channel support the dissemination of the courses to target groups of learners. For instance, an information webinar was held in June 2022 for the release of the *Inequality monitoring in HIV, tuberculosis and malaria* course. The webinar, which involved the participation of the Global Fund to Fight AIDS, Tuberculosis and Malaria, was targeted to: WHO and Global Fund technical staff; Ministry of Health staff from Member States, including monitoring and evaluation officers and disease programme experts; staff of HIV, tuberculosis and malaria organizations such as UNAIDS, International AIDS Society, StopTB Partnership and Roll Back Malaria Partnership; and researchers, faculty members and students from academic institutions. A launch event for the health inequality monitoring foundations series is planned for September 2022. 

The courses do not fully meet the needs of all potential learners; there remain gaps to be filled through other resources and delivery methods. Further efforts to evaluate the impact of the courses and opportunities for improvement will be undertaken as the courses roll out. Periodic updates to the courses are planned, informed by learner feedback and the course discussion forums on the OpenWHO platform. Anonymized learner characteristics will be tracked to assess the geographic and demographic reach of the courses. To support the overarching aim of the Health Inequality Monitoring team, to strengthen capacity for health inequality monitoring among Member States, the contents of courses may help to inform – or be integrated into – the subsequent development of other health equity and population health monitoring eLearning courses. This may include courses that are part of the new WHO Academy [25] or other massive online open courses (MOOC) providers, developed in conjunction with collaborators across WHO or other academic, governmental or non-governmental entities.

These eLearning courses serve as an additional resource to help support health inequality monitoring in areas where it is not done or could be strengthened. A fundamental concern going forward relates to the availability of disaggregated data of adequate quality across domain areas – as this is the basic requirement for inequality monitoring [26]. Incorporating inequality monitoring into country health information systems, moreover, has other requirements such as political will, financial resources, multisectoral engagement and multistakeholder partnerships [27]. Health inequality monitoring should be integrated into the development, implementation and evaluation process of policies, programmes and practices to promote sustained equity-oriented changes.

## Data Availability

The Health Inequality Monitoring eLearning channel is available at https://openwho.org/channels/inequality-monitoring. The other WHO tools and resources for health inequality monitoring referred to in this article are available via the Health Inequality Monitor at https://www.who.int/data/inequality-monitor.

## Appendix 1

### Health inequality monitoring foundations courses content

Course name: Overview

Available from: https://openwho.org/courses/inequality-monitoring-overview

- Module 1: Key concepts and terminology
- Module 2: Situating health inequality monitoring
- Module 3: Cycle of monitoring
- Module 4: WHO resources

Course name: Data sources

Available from: https://openwho.org/courses/inequality-monitoring-data-sources

- Module 1: Household surveys
- Module 2: Administrative data sources
- Module 3: Civil registration and vital statistics systems
- Module 4: Censuses
- Module 5: Data source mapping
- Module 6: Data linking

Course name: Health data disaggregation

Available from: https://openwho.org/courses/inequality-monitoring-health-data-disaggregation

- Module 1: Health indicators and dimensions of inequality
- Module 2: Measuring dimensions of inequality
- Module 3: Categorizing dimensions of inequality
- Module 4: Reporting disaggregated health data

Course name: Summary measures of health inequality

Available from: https://openwho.org/courses/inequality-monitoring-summary-measures

- Module 1: Defining characteristics of summary measures
- Module 2: Simple summary measures
- Module 3: Complex summary measures
- Module 4: Interpreting and reporting summary measures

Course name: Reporting

Available from: https://openwho.org/courses/inequality-monitoring-reporting

- Module 1: Purpose and audience
- Module 2: Scope and technical content
- Module 3: Data presentation approaches
- Module 4: Best practices

## Appendix 2

### Topic-specific applications of health inequality monitoring courses content

Course name: Inequality monitoring in HIV, tuberculosis and malaria

Available from: https://openwho.org/courses/inequality-monitoring-hiv-tb-malaria

- Module 1: Determine scope of monitoring
- Module 2: Obtain data
- Module 3: Analyse data
- Module 4: Report results
- Module 5: Knowledge translation

Course name: Inequality monitoring in immunization

Available from: https://openwho.org/courses/inequality-monitoring-immunization

- Module 1: Determine scope of monitoring
- Module 2: Obtain data
- Module 3: Analyse data
- Module 4: Report results
- Module 5: Knowledge translation

## Abbreviations

ADDIE: Analysis-Design-Development-Implement-Evaluate
HEAT: Health Equity Assessment Toolkit
TOT: Training of Trainers
WHO: World Health Organization

## Footnotes

1 The content and format of health inequality monitoring capacity building workshops have varied: while some had a country or thematic focus, others were conducted within a select region or for a group of countries. Workshop participants have included: participants working in countries’ ministries of health, statistical offices, academia and public health institutes; WHO collogues working in headquarters, regional and countries offices; and participants from other global health organizations like Gavi, the Vaccine Alliance and the Global Fund. Prior to enrolling in the workshop, all participants were required to have the ability to interpret, summarize and report data, and a working knowledge of health information.
